# Advancements in Individual Dental Implants: A State-of-the-Art Review of Materials and Technologies

**DOI:** 10.3390/ma19091732

**Published:** 2026-04-24

**Authors:** Monika Lukomska-Szymanska, Mateusz Radwanski, Michal Leski, Aftab Ahmed Khan, Jukka P. Matinlinna

**Affiliations:** 1 Department of General Dentistry, Medical University of Lodz, 251 Pomorska Str., 92-213 Lodz, Poland; 2 Department of Endodontics, Medical University of Lodz, 251 Pomorska Str., 92-213 Lodz, Poland; mateusz.radwanski@umed.lodz.pl (M.R.); michal.leski@umed.lodz.pl (M.L.); 3 Dental Health Department, College of Applied Medical Sciences, King Saud University, Riyadh 11433, Saudi Arabia; aftkhan@ksu.edu.sa; 4 Biomaterials Science, Division of Dentistry, The University of Manchester, Manchester M13 9PL, UK; jpmat@hku.hk; 5 School of Dentistry, The University of Jordan, Amman 11942, Jordan

**Keywords:** customized implants, manufacturing technologies, PEEK, titanium, zirconia

## Abstract

Objective: This narrative review synthesizes current evidence on materials and manufacturing technologies for customized dental implants, highlighting their comparative advantages and limitations. Methods: A structured literature search (December 2024–January 2025) was conducted using PubMed, Web of Science, Scopus, and Google Scholar. Peer-reviewed English-language articles (mainly 2015–2025) addressing implant materials, manufacturing methods, and surface modifications were included. Data were critically analyzed and thematically organized without meta-analysis. Results: Digital workflows are advancing implantology toward patient-specific solutions. Subtractive manufacturing (SM) ensures high precision and surface quality but is limited by material waste and geometric constraints. In contrast, additive manufacturing (AM) enables complex, porous, and customized designs, though often requires post-processing. Titanium and its alloys remain the gold standard due to strength and biocompatibility, while TiZr and β-type alloys may reduce stress shielding. Zirconia offers aesthetic benefits but is brittle, whereas PEEK shows favorable elasticity but limited bioactivity. Surface modifications enhance osseointegration and long-term performance. Conclusions: Combining digital workflows with SM and AM supports development of optimized, patient-specific implants. While titanium dominates clinical use, emerging materials offer specific advantages. Further clinical validation and standardization are required.

## 1. Introduction

Driven by breakthroughs in intraoral scanning, digital imaging, computational design, and additive manufacturing, the field of implant dentistry is undergoing a fundamental shift. These technologies are enabling a significant move beyond standardized implants, paving the way for patient-specific individual designs that are optimized for superior biological integration [1]. Today, dental implants are available in a variety of designs, fixture shapes and dimensions (diameter, length) to accommodate different clinical scenarios and anatomical conditions [2,3]. Nevertheless, anatomical heterogeneity, compromised bone, and aesthetic demands have spurred interest in personalized implant geometries instead of conventional dimensional ranges. Traditional CAD-CAM (computer aided design and computer aided manufacturing) milling may not provide an ultimate answer. In conjunction with geometrical optimization, the development of implant materials has evolved from commercially pure titanium (Ti) and Ti-6Al-4V to advanced Ti alloy longevity [4,5].

Customized implants can better adapt to the geometry of the extraction socket, which may contribute to improved primary stability and support of the peri-implant soft tissue profile; however, this does not eliminate the risk of buccal bone resorption [6]. To obtain a personalized dental implant, data are first acquired using cone beam computed tomography (CBCT) before surgery. This imaging modality provides high-resolution scanned 3D information about the patient’s maxillofacial structures, allowing the precise assessment of bone quality, its volume, density, and the spatial relationship to critical anatomical landmarks. The CBCT data are then converted into digital files that can be processed further with specialized computer-aided design software. Within this virtual environment, a 3D model of the patient’s jaw is reconstructed, on which both the implant body and its abutment can be individually designed to achieve an optimal fit and functional alignment. Given that, the finalized design is subsequently transferred to computer-aided manufacturing systems, where it is fabricated using either additive or subtractive manufacturing techniques [7].

An alternative approach involves direct intraoperative or preoperative digitization of the extracted tooth root using high-precision laser scanning. This technique enables the creation of a root-analogue implant that mimics the natural morphology of the patient’s tooth, thereby improving primary stability and reducing surgical trauma. To further enhance retention, macroretainers can be incorporated into the implant surface design, such as ridges, grooves, or undercuts, which mechanically interlock with the alveolar socket following extraction [8].

This narrative review provides a state-of-the-art synthesis of individualized dental implants by, firstly, explicitly addressing the gap left by earlier reviews that treated material properties, fabrication technologies, and biological performance in isolation and, instead, integrating them into a single, end-to-end digital workflow from CBCT/intraoral data acquisition through CAD, CAM programing, manufacturing (subtractive and multiple additive routes) and post-processing to clinical delivery. It is further advancing the field by systematically comparing and contrasting subtractive and additive manufacturing specifically for customized implants, relating each technology to its ability to realize patient-specific macro- and micro-geometries, internal architectures, and stiffness gradients. In parallel, it is linking the key material classes (i.e., Ti and advanced Ti alloys, zirconia, PEEK and some other composites) to their customization potential, biomechanical behavior versus bone, current clinical evidence, and surface pretreatment options, using structured tables and figures to provide a clinically oriented, comparative framework that was previously missing. Therefore, the aim is to provide a comprehensive overview of current developments, materials, and manufacturing technologies used in the design and fabrication of customized dental implants.

## 2. Materials and Methods

### 2.1. Review Design

This narrative review is synthesizing current knowledge on materials and manufacturing technologies for customized dental implants. Due to the topic’s broad, interdisciplinary scope, including digital workflows, additive/subtractive manufacturing, metallic/ceramic biomaterials, and surface modifications, a narrative approach was chosen over quantitative meta-analysis to deliver a comprehensive, clinically oriented overview.

### 2.2. Search Strategy

A systematic literature search was conducted in December 2024 and updated in January 2025 across PubMed, Web of Science, Scopus, and Google Scholar. The strategy integrated keywords and MeSH terms across three domains, i.e., customized implants (“customized dental implant,” “patient-specific implant,” “individualized implant,” “root analogue implant”), manufacturing technologies (“additive manufacturing,” “3D printing,” “selective laser melting,” “electron beam melting,” “fused deposition modeling,” “subtractive manufacturing,” “CAD/CAM,” “CNC milling”), and biomaterials (“titanium,” “Ti-6Al-4V,” “TiZr,” “β-titanium alloy,” “zirconia,” “Y-TZP,” “PEEK,” “surface modification,” “osseointegration”), using Boolean operators (AND, OR). Duplicates were removed, and reference lists of included articles and relevant reviews were hand-searched for additional studies.

### 2.3. Inclusion and Exclusion Criteria

Articles were included if they were original research, clinical trials, observational studies, systematic reviews, or narrative reviews from peer-reviewed journals addressing materials for dental implants (e.g., titanium alloys, zirconia, PEEK), additive/subtractive manufacturing of customized implants, or surface pretreatment methods, published in English with no lower date limit (prioritizing 2015–2025). Articles were excluded if they were conference abstracts, editorials, opinion pieces, non-peer-reviewed sources, focused solely on removable prostheses, orthodontic appliances, or non-dental implants, or if full text was unavailable.

In addition to database searches, reference lists of selected articles were manually screened for further relevant publications.

Given the narrative nature of this review, no formal meta-analysis or quantitative synthesis was performed. Instead, the literature was critically analyzed and thematically organized into key domains: (i) manufacturing technologies (subtractive and additive), (ii) materials for customized implants, and (iii) surface modification strategies and biological performance. Emphasis was placed on recent advances in additive manufacturing, titanium alloys, and material–processing–biological interactions to capture current trends and clinical directions.

## 3. Results

### 3.1. Manufacturing Technologies

#### 3.1.1. Subtractive Technology

Subtractive machining technology uses turning, milling, grinding, or other mechanical approaches to remove material from a specific material to form a desired shape. For dental implants, subtractive manufacturing has long been the standard approach, as casting Ti is just too cumbersome. However, other techniques are rapidly emerging. It is noteworthy that subtractive manufacturing provides superior dimensional accuracy, which also conveniently accepts the use of well-known, biocompatible materials and depends on existing and tested workflows. The suitable material is chosen, based on the clinical application: titanium alloy (often Ti-6Al-4V), layered or monolithic zirconium dioxide (ZrO_2_), or polyetheretherketone (PEEK) as presented in Table 1 [9].

The process of implant production starts with acquiring digital information from CBCT scans to evaluate the bone quality and structure and intraoral scans to record the soft and hard tissues in the oral cavity. Next, specialized CAD software is used to design the digital implant with both functional (fit, load) and aesthetic requirements (such as surrounding mucosa and adjacent teeth). Then, the design is converted into a CAM program that generates precise tool paths, which allow the removal of material in controlled condition to achieve the desired shape. After milling or turning, the finishing operations include visible surface defect polishing and/or surface pretreatment, i.e., grit-blasting or acid etching to promote bone on-growth. The final step involves cleansing from debris, sterilization, packaging and delivery to the dental clinic or surgery.

One of the real clinical advantages of subtractive machining is the high precision and accuracy achieved through the computer numerical control (CNC), which allows fabrication of components for prosthodontics with high precision and accuracy and reproducible results [10]. Additionally, subtractive machining provides excellent surface quality and fine detail resolution, reducing extensive post-processing. The process is also time-efficient for certain applications, especially when mass production or standardized parts are required, as well as highly versatile, being compatible with a wide range of biocompatible prosthodontic materials such as titanium, zirconia, and CoCr alloys [11].

Yet, this technique is limited by the fact that a cutting tool can only reach spaces in a straight line, and the high-speed cutting process creates vibration and forces that may damage delicate features. These restrictions make it somewhat difficult to create intricate internal structures, porosity gradients, or nature-inspired lattice designs—features now being developed to improve bone integration. Also, a significant amount of waste is generated from the milling block material that may be difficult to recycle. This occurs because substantial portions of the material must be removed to achieve the desired shape. Consequently, it reduces both sustainability and cost-effectiveness. Moreover, the material cannot be reused in its original form but would require tedious processing. These limitations become particularly relevant when manufacturing patient-specific implants requiring anatomical conformity or stiffness optimization. In addition, the equipment costs for high-precision CNC machines and the need for skilled operators may easily increase overall production expenses. Tool wear during milling or grinding also affect surface integrity and dimensional accuracy over time [12].

#### 3.1.2. Additive Manufacturing Technology (AM)

AM, commonly known as 3D-printing, represents a paradigm shift in implant production and digital dentistry; today enabling design-driven fabrication rather than geometry is constrained by tooling limitations. Unlike traditional subtractive manufacturing, AM builds components layer by layer (even 0.1 mm thin) directly replicating computer-aided design (CAD) models. This approach allows precise fabrication of complex geometries and patient-specific implant designs that would be difficult or impossible to achieve using conventional methods. In dental implantology, AM typically utilizes biomaterials such as titanium alloys (e.g., Ti-6Al-4V), CoCr, ceramics [e.g., zirconia or alumina (Al_2_O_3_)], and polymers, e.g., PEEK or even poly(methyl methacrylate) [PMMA], and certain bioactive composites [13].

The production workflow begins with obtaining patient-specific data through intraoral scanning or CT imaging, and a 3D implant model is designed using CAD software. Importantly, AM enables integration of biomechanical design features such as surface structures, topology-optimized geometries, and stiffness gradients aimed at improving even load distribution and bone ongrowth. The model is then printed layer by layer (each ca, 0.1 mm) from biocompatible powder materials utilizing AM techniques (e.g., selective laser melting) [6,7]. After 3D-printing, the implant undergoes post-processing steps such as heat treatment to relieve internal residual stresses and surface modification to improve osseointegration. Metallic implants generally require heat treatment and often a surface modification. Ceramic implants require sintering and sometimes surface modification whereas polymeric implants usually undergo a surface modification but no heat treatment for annealing. Afterwards, supports are removed, and the surface is polished, grit-blasted, or etched to achieve the desired roughness for bone attachment. Finally, the implant is thoroughly cleaned from impurities and debris and sterilized to eliminate any biological contaminants [14].

Beyond manufacturing efficiency, the key advantage of AM lies in its ability to control internal architecture and surface topography during fabrication, allowing simultaneous optimization of mechanical and biological performance. It is noteworthy that AM technology guarantees high precision, consistent results, and efficient production by minimizing flaws. By requiring less raw material milling blocks and enabling their reuse, AM is considered both cost-effective and environmentally sustainable [15].

Several other AM technologies are commonly used in dentistry, but today the most popular in implant fabrication is powder bed fusion (PBF) or metal extrusion (MEX). Powder bed fusion includes selective laser sintering (SLS), direct metal laser sintering (DMLS), selective laser melting (SLM), and electron-beam melting (EBM) [16]. These techniques use high-power lasers, or electron beams to melt small powder particles into thin layers, layer by layer [6,7,16].

##### Selective Laser Sintering (SLS) and Selective Laser Melting (SLM)

SLS binds high-melting-point metal or non-metal powders by melting a low-melting metal or binder [17]. In contrast, SLM uses a high-power laser with a small spot to quickly and completely melt and sinter metal powder and requires a higher laser power density than that used in SLS [7]. From a customization standpoint, SLM facilitates meticulous regulation of porosity and microarchitecture, potentially improving osseointegration and minimizing elastic modulus discrepancies with adjacent bones. Furthermore, although SLM parts present good mechanical strength, they can suffer from high internal stresses caused by induced thermal gradients during processing, requiring additional heat treatment. In the SLS process, selected polymers such as polyamides (PA), polystyrene (PS), thermoplastic elastomers (TPE), and polyaryletherketones (PAEK) are the most popular [16]. However, not all of them would be as viable as a dental implant material.

In SLM by and large, some commonly manufactured metallic objects include pure metals such as Ti, Cu, and W, as well as metal alloys like Ni-based superalloys, stainless steel, tool steel, CoCr, and Al alloys [16].

##### Electron Beam Melting (EBM)

EBM is also a powder bed melting technology. The production principles using EBM and SLM are similar, but the heat sources are different. EBM uses an electron beam generated by accelerating electrons in an ultrahigh vacuum as the heat source, while SLM applies a high-power monochromatic laser beam to melt and fuse the metal powder [18]. Compared to laser-based technologies, EBM has quicker build rates and less residual stress because it uses higher processing temperatures, which could affect the microstructure and fatigue behavior of the implant [19]. Moreover, EBM has many advantages, such as high energy utilization, no reflections, high power density and convenient focusing, which can be used to fabricate implants [20]. On the other hand, there are disadvantages which include a relatively rough surface finish, lower resolution and inferior dimensional accuracy compared to laser-based methods, and the need for operation in a high-vacuum environment, which increases cost and limits the build size [20]. The process is also restricted to conductive materials, and it requires complex and expensive production equipment. In addition, often post-processing is necessary to achieve the desired surface finish, quality and precision. This technology employs titanium and CoCr alloys.

##### Fused Deposition Modelling (FDM)

**Figure 1:**
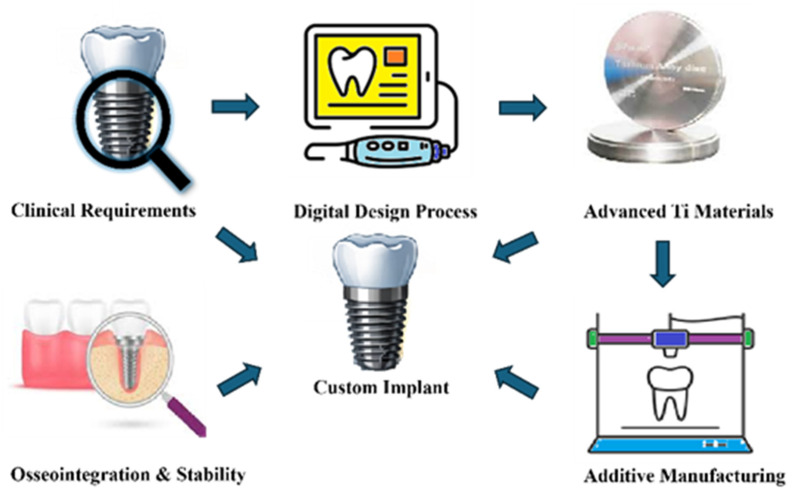
Digital workflow for customized dental implant fabrication, illustrating the integration of clinical diagnosis, CBCT-based digital planning, CAD, material selection, additive/subtractive manufacturing, and final implant placement.

In fused deposition modelling technology, which belongs to the group of material extrusion (MEX), the thermoplastic material is heated, melted and extruded to produce filaments [8]. After cooling, the filamentous material is joined layer by layer, creating a thin cross-section, and finally the layers are superimposed on each other to form a three-dimensional structure. Although largely utilized for prototyping or experimental implant concepts, FDM provides quick iteration of customized designs and the creation of polymer-based or composite implants [21]. FDM offers advantages such as low cost, ease of use, and the ability to produce complex geometries with a wide range of thermoplastic materials. It is well-suited for rapid prototyping and functional testing. However, FDM presents with certain disadvantages including relatively low resolution, visible layer lines, weaker mechanical properties (cohesion) due to interlayer bonding, limited material choices compared to other additive manufacturing methods, and often requires post-processing to improve surface finish and final strength [22]. On the other hand, FDM technology can process a variety of materials, including polymers such as polylactic acid (PLA), polyamide (nylon), and PEEK, as well as some ceramics. In many biomedical applications, ceramics are typically incorporated as polymer–ceramic composites—for example, PLA/HA (hydroxyapatite), PEEK/HA, or PEEK/ β-TCP (β-tricalcium phosphate)—rather than used in their pure form [23,24]. The complete digital workflow for customized implant fabrication is illustrated in Figure 1.

### 3.2. Materials Used for Customized Dental Implants

In dental implantology, with regard to superior mechanical properties, including high strength, modulus, and excellent resistance to wear and corrosion, selected metals and their alloys are favored in the biomedical field (Table 2).

#### 3.2.1. Ti Alloys

The most investigated and clinically popular powdered materials for customized dental implants are Ti alloys: commercially pure (c.p. Ti) grade I-IV and Ti-6Al-4V (grade V) [25]. Commercially pure Ti is favored for its excellent biocompatibility because it forms a stable, inert, and tightly adherent extremely thin oxide layer (TiO_2_) on its surface when exposed to air or body fluids, providing corrosion resistance and enabling direct osseointegration [26]. The higher c.p. Ti grades (III and IV) provide greater tensile strength (about 450 MPa- Grade III and up to 550 MPa Grade IV) compared to the more ductile lower grades I (around 240 MPa) and II (about 350 MPa). On the other hand, Ti-6Al-4V (Grade V) offers superior tensile strength (appr. 900–950 MPa) and fatigue resistance (up to 550 MPa), making it particularly suitable for implants subjected to higher functional loads [27].

**Figure 2:**
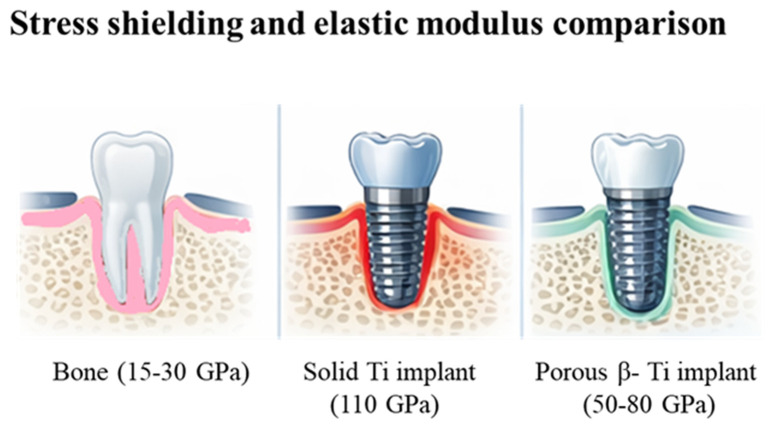
Schematic comparison of load transfer and elastic modulus mismatch. **Left:** natural tooth and surrounding bone (15–30 GPa). **Middle**: solid titanium implant (~110 GPa) demonstrating stress shielding. **Right**: porous or β-type titanium implant (50–80 GPa) suggesting improved biomechanical compatibility.

Both materials exhibit favorable surface characteristics with naturally forming oxide layer titanium dioxide that supports bone ongrowth. Their long history of clinical success has firmly established them as the benchmark in implant dentistry. C.p. Ti has lower tensile and fatigue strength, limiting its use in high-load applications, while Ti-6Al-4V, though stronger, is less ductile and may be more prone to a brittle fracture [27]. Nonetheless, both materials exhibit higher modulus of elasticity than bone, potentially causing stress shielding and bone resorption. This biomechanical mismatch and its potential consequences are illustrated schematically in Figure 2. This may require surface finishing to optimize integration and reduce bacterial colonization in situ. Additionally, it is reported that Ti-6Al-4V can release alloying ions under extreme conditions, and both materials involve higher costs and manufacturing complexity compared to other biomaterials. For sure, one cannot even exclude the rare Ti allergy (estimated in ca. 0.6% in the general population) [28]. Beyond conventional Ti-6Al-4V, alternative titanium alloys can be used for additive manufacturing of customized dental implants. In particular, TiZr alloys, including Ti-15Zr systems for narrow implants such as Roxolid^®^, exhibit superior strength and enhanced corrosion resistance relative to commercially pure titanium, facilitating the fabrication of smaller-diameter implants without compromising mechanical performance [29]. Nevertheless, their adoption in powder bed fusion (PBF) processes remains limited compared with Ti-6Al-4V, owing to the lack of standardized powder feedstocks and established processing parameters. Concurrently, metastable β-type titanium alloys, including TiMo, TiNb, and related systems, are under investigation for their promising reduced elastic modulus, approaching that of cortical bone (appr. 50–80 GPa). This feature minimizes stress shielding and enhances biomechanical compatibility, particularly when integrated with porous or lattice architectures enabled by AM [30].

#### 3.2.2. Zirconia

Zirconia, particularly yttria-stabilized tetragonal zirconia polycrystal (Y-TZP), represents the second most investigated material in dental implantology, primarily due to its excellent biocompatibility, favorable mechanical properties, and superior aesthetics [31]. Unlike metallic implants, zirconia exhibits a tooth-like color, making it especially suitable in cases where high aesthetic demands are required, such as in anterior regions. In addition, zirconia demonstrates low plaque accumulation and reduced bacterial adhesion compared to Ti due to its surface chemistry characteristics [32]. Allergies to ceramics are unknown. The polished zirconia surface is highly inert and hydrophilic making it less favorable for bacterial colonization [32]. Y-TZP can be polished to a very smooth finish, minimizing microscopic irregularities where bacteria typically accumulate, adhere and form biofilm. The gum tissue responds well to the polished ZrO_2_ surface around the implant neck, promoting stable soft tissue attachment and reducing inflammation. This smooth cervical surface supports a tight and healthy interface between the implant and surrounding tissue, helping prevent gum recession and maintain long-term aesthetics and expected implant function. However, despite its high flexural strength (from 900 to 1200 MPa) and fracture toughness (between 5 and 10 MPa m^1/2^), zirconia remains brittle (with fracture strain <1%) in contrast to Ti and is therefore more susceptible to fracture under impact and excessive loading [33].

To overcome these limitations, current research has focused e.g., on optimizing yttria content (3–5 mol% Y_2_O_3_), developing partially stabilized zirconia formulations, and applying surface pretreatments to enhance both mechanical reliability and biological performance [34,35]. While such surface modifications can improve osseointegration and soft tissue attachment, they must be carefully controlled, as roughening or introducing micro-notches can create stress concentrators in the zirconia matrix, reduce fracture toughness, and lower fatigue resistance [35,36]. Other disadvantages include still limited long-term clinical data compared to Ti, difficulty in intraoral adjustments due to its hardness, cumbersome unscrewing (removal), sensitivity to surface defects or micro-cracks that can act as stress concentrators, and challenges in manufacturing complex geometries without compromising mechanical reliability [37]. Additionally, phase transformation or low-temperature degradation over time may slightly reduce strength and toughness in humid environments [38]. Even tough long-term clinical evidence is still emerging, one-piece ZrO_2_ implants are increasingly considered a viable metal-free alternative for patients with titanium hypersensitivity or for cases demanding superior aesthetics [39].

#### 3.2.3. Polyetheretherketone (PEEK)

In recent times, polyetheretherketone is considered as a potential implant material used in 3D-printing technology. PEEK is an aromatic semicrystalline tan colored polymer with good chemical stability, high melting point (343 °C), X-ray transmission, high strength and high temperature resistance (up to ~250 °C) [40]. The elasticity of PEEK (~3–4 GPa) is lower than that of titanium (~110 GPa for c.p. Ti, ~110–120 GPa for Ti-6Al-4V), and closer to cortical bone (~10–30 GPa); thus it may reduce the peri-implant bone loss and enhance implant integration [41,42]. The main challenge in case of this material is low surface free energy (~20–25 mN/m); therefore the strategies to enhance the biological activity are applied, e.g., plasma surface treatment or sulfonation [43]. FDM and SLS technologies can be used to print PEEK implants. However, as such PEEK is not inherently osteoconductive.

### 3.3. Surface Pretreatment

A critical factor influencing clinical success is the surface topography of the dental implant, particularly its roughness profile and porosity. Implant surfaces can be modified, chemically (e.g., acid etching), or physicochemically (e.g., deposition of bioactive coatings such as hydroxyapatite) to enhance their biological performance [44,45].

#### 3.3.1. Mechanical Pretreatments

Mechanical pretreatments, such as grit-blasting, involve bombarding the implant surface with abrasive particles (e.g., aluminum oxide, titanium oxide, silicon carbide), creating micro-roughness that dramatically increases the surface area for bone contact and promotes mechanical interlocking with the surrounding tissue [46,47]. Ti-based implants with sandblasted and acid-etched (SLA) or sandblasted, large-grit, and acid-etched (SLActive™) surfaces exhibit high resistance to mechanical damage, maintaining their microtopography even under high implant fixture insertion torque. In contrast, plasma-sprayed hydroxyapatite (HA) coatings, although highly bioactive, are more susceptible to delamination, cracking, or partial detachment during implant placement, especially in dense bone, due to their natural brittle nature and limited adhesion strength to the metallic substrate [48]. Zirconia implants with polished or laser-textured surfaces show good mechanical stability but may potentially develop microcracks if excessive force is applied. For PEEK-based implants, thin bioactive coatings such as HAp, TiO_2_, or Ca–phosphates deposited by plasma spraying, magnetron sputtering, or sol–gel methods enhance osseointegration, but their adhesion strength remains initially lower than coatings on metallic substrates [49].

Mechanical surface pretreatments can be applied to implants made from Ti and its alloys (c.p. Ti, Ti-6Al-4V), Y-TZP, and CoCr-alloys. Great care must be taken with brittle materials like zirconia to avoid initial flaws, such as micro-cracks, while very thin or low-strength ceramics and coatings are generally unsuitable for such treatments. These procedures create micro-roughness that enhances bone apposition, osseointegration and mechanical interlocking with surrounding tissue.

The aluminum oxide (Al_2_O_3_) powder is the gold standard for pretreatment of titanium implants with the particle size typically ranging from 25 to 250 µm, with coarser particles producing deeper roughness and finer particles producing smoother microtextures. The size, shape, and hardness of the abrasive particles, as well as the pressure and duration of blasting, can be carefully controlled to produce surfaces with different roughness profiles tailored for specific clinical applications. The typical grit-blasted dental implants achieve R_a_ values of 1–2 µm using particle sizes optimized for the material, pressures of 2–4 bar (200–400 kPa), and controlled blasting angles and distances to ensure uniform micro-pits without damaging the surface [50]. Additionally, mechanical pretreatments can remove surface contaminants and oxide layers, exposing a fresh titanium surface that further improves biological response [51,52,53,54]. Beyond promoting bone growth, the increased surface roughness also helps distribute mechanical loads more evenly across the implant-bone interface, potentially reducing stress shielding and peri-implant bone resorption. Recently, even cavitation erosion as an osteoinductive process on Ti has been suggested [55].

#### 3.3.2. Chemical Pretreatments

Chemical methods, including acid etching or anodization, selectively remove portions of the implant surface to create a micro- and nano-scale topography that is highly conducive to bone tissue integration. These surface pretreatments are primarily applied to titanium and its alloys (c.p. Ti, Ti-6Al-4V) and are generally unsuitable as such for zirconia or other brittle ceramics, which may be damaged by aggressive chemical exposure [56]. Acid etching, often performed with strong mineral acids (or their blends) such as HF, HCl, H_2_SO_4_ or HNO_3_, produces a topographic network of pits and microgrooves that increase the surface area, deepen the profile, and provide physical anchorage points for osteoblasts. This roughened surface significantly improves cell adhesion, spreading, and proliferation, which are essential for the early stages of osseointegration. In addition to creating topographical features, chemical pretreatments enhance the surface free energy and wettability, allowing biological fluids, proteins, and growth factors to more effectively interact with the implant, promoting faster bone healing [57]. Anodization, a controlled electrochemical process, can further modify the thickness, porosity, and composition of the TiO_2_ layer, improving corrosion resistance while also supporting cellular activity [58]. These nano-scale modifications not only stimulate osteogenic cell responses but also influence protein adsorption and signaling pathways critical for bone remodeling. These methods can be combined with mechanical treatments, such as sandblasting, to produce synergistic effects, resulting in micro–macro roughened surfaces that maximize both mechanical interlocking and biological activity [58].

#### 3.3.3. Physicochemical Modifications

Physicochemical modifications combine both chemical and physical principles to further enhance surface bioactivity; this includes the deposition of thin bioactive coatings (bioceramics) such as hydroxyapatite (HA), calcium phosphate, or other Ca-based compounds, which mimic the mineral composition of natural bone and may stimulate faster bone formation [59,60]. They are primarily applied to Ti and its alloys (c.p. Ti, Ti-6Al-4V) and, to a lesser extent, Y-TZP. These thin coatings not only provide a favorable chemical environment for osseointegration but can also act as carriers for growth factors or anti-microbial agents, improving both the speed and quality of bone-implant osseointegration [55,61]. Nevertheless, their mechanical resistance is limited compared to the underlying titanium or zirconia substrate. These ceramic coatings are generally brittle and relatively thin, so they can delaminate, crack, or wear under high functional loads, impact, or micromotions at the bone–implant interface. Therefore, while surface modifications improve biological interactions, they do not significantly enhance the bulk mechanical strength of the implant, and care must be taken during placement and loading to avoid coating damage [62].

Additionally, advanced surface alteration techniques such as plasma spraying, magnetron sputtering, or sol–gel deposition allow precise control over coating thickness, porosity, and crystallinity, tailoring the surface to meet specific biomechanical and biological requirements [61,63,64]. They are primarily applied to titanium and its alloys (c.p. Ti, Ti-6Al-4V) and, in some cases, Y-TZP. By combining these approaches, implant surfaces can achieve an optimal balance of mechanical interlocking, chemical signaling, and biological compatibility, which is essential for long-term stability and clinical success.

## 4. Discussion

The transition from traditional SM to AM marks a fundamental evolution in implant dentistry, moving the field away from standardized dimensional ranges toward truly patient-specific, design-driven solutions [6,7]. While SM, such as CNC milling, provide superior dimensional accuracy and rely on well-established workflows, they are inherently limited by substantial material waste and straight-line cutting constraints that prevent the creation of complex internal structures [10,12]. Conversely, AM techniques, particularly powder bed fusion methods like SLM and EBM, enable the precise layer-by-layer fabrication of tailored geometries [6,18]. This design freedom allows for the integration of topology-optimized features and porosity along with minimizing the elastic modulus mismatch between the implant and the surrounding alveolar bone, thereby reducing the risk of stress shielding [4,5].

The success of these customized geometries remains heavily dependent on the selection of appropriate biomaterials. Titanium alloys, specifically Ti-6Al-4V, continue to serve as the clinical benchmark due to their exceptional tensile strength, fatigue resistance, and predictable osseointegration [26,30]. However, the inherent stiffness and clinical limitations of conventional Ti-6Al-4V have driven the exploration of advanced Ti alloys designed to optimize both mechanical resilience and biomechanical compatibility. Specifically, TiZr alloys exhibit superior strength and corrosion resistance, making them highly advantageous for the fabrication of narrow-diameter implants in space-restricted regions without sacrificing structural integrity [29]. Concurrently, metastable β-type Ti alloys address the critical issue of stress shielding by offering a significantly reduced elastic modulus that more closely mimics natural cortical bone [27,30]. When synergized with the porous architectures enabled by additive manufacturing, these advanced alloys represent a crucial step forward in developing patient-specific implants that ensure long-term stability and bone preservation [4,5,6]. Beyond titanium, for patients presenting with metal hypersensitivity or demanding high aesthetic outcomes in anterior regions, zirconia (Y-TZP) serves as a highly biocompatible, tooth-colored alternative with notably low plaque affinity [28,31,35]. Yet, zirconia’s brittleness and susceptibility to micro-cracks under high impact forces necessitate careful case selection and design constraints [33,38]. Meanwhile, emerging polymeric materials like PEEK offer highly favorable elastic properties, though their inherent lack of osteoconductivity requires mandatory surface modifications to achieve adequate biological integration [40,42,43].

Ultimately, regardless of the primary manufacturing technology or base material utilized, surface topography acts as a decisive factor in achieving long-term clinical success.

Excessive porosity or manufacturing defects may act as unwanted stress concentrators, increasing the risk of fatigue fracture under cyclic occlusal loading, while insufficient mechanical strength can lead to deformation or implant failure, particularly in high-load posterior regions. In addition, weak adhesion of coatings or inadequate surface integrity may compromise osseointegration, promote bacterial colonization, and accelerate peri-implant bone loss, ultimately reducing implant stability, longevity, and overall clinical success [46].

Modifying the customized implant surface through mechanical, chemical, or physicochemical pretreatments is absolutely essential and imperative to enhance cellular adhesion and optimize biological performance [46,56]. Techniques such as grit-blasting and acid etching create a vital micro-roughness that facilitates initial bone on-growth, improves mechanical interlocking, and increases the surface free energy for favorable protein interactions [47,48,57]. Furthermore, advanced physicochemical modifications, including the deposition of bioactive bioceramic coatings, show significant promise in accelerating osseointegration by mimicking the natural mineral composition of bone [59,60]. Moving forward, the most successful patient-specific implants will likely rely on an integrated approach that simultaneously combines customized AM geometries, biomechanically optimized materials, and tailored surface topographies to address both anatomical and biological challenges [1,6].

Nonetheless, several limitations of this study warrant acknowledgment. Firstly, the narrative nature of the study, lacking a systematic methodology, may introduce selection bias. Secondly, the existing literature exhibits heterogeneity in study design, materials, and reported outcomes, complicating direct comparisons. Notably, data on customized implants and novel manufacturing techniques remain limited, particularly concerning long-term clinical performance. Furthermore, the reported properties of certain materials, such as TiZr alloys, may vary based on experimental conditions, including manufacturing methods, surface treatments, and testing protocols. These variations can affect the reported mechanical and biological performance, necessitating careful consideration when interpreting results.

Consequently, we are not yet there: future research should prioritize well-designed and defined clinical studies with extended follow-up periods to more accurately assess the survival and success rates and complications. Additionally, the development of standardized protocols for additive manufacturing processes and surface treatments is essential to facilitate reliable comparisons. Further investigations should also explore emerging and innovative materials, including advanced titanium alloys, bioactive composites, and functionally graded structures, with a particular focus on their long-term clinical performance and reliability.

## 5. Conclusions

Dental implantology is shifting toward digitally driven, patient-specific solutions enabled by advanced imaging, individualized design, and additive manufacturing. Titanium and its alloys remain the clinical gold standard due to their proven performance; however, additive manufacturing allows improved control over implant geometry, porosity, and mechanical properties, supporting better biomechanical compatibility.

Emerging materials such as TiZr and β-type Ti alloys show potential to reduce stress shielding and improve implant–bone interaction, although their broader clinical use is still limited by the need for further validation and process optimization. The findings of this review highlight that successful implant design depends on the integration of material selection, manufacturing technology, and surface engineering within a unified workflow.

From a practical perspective, these developments support the transition toward more personalized and functionally optimized implants. Future research should focus on long-term clinical outcomes, particularly for newer materials, as well as on standardization of additive manufacturing processes and further development of biofunctional and structurally optimized implant systems.

## Figures and Tables

**Table 1:** Materials used in subtractive manufacturing technology of dental implants.

Material	Form/Grades	Typical Indications
*Metallic:* Commercially pure titanium (c.p. Ti)	Grade I–IV	Endosseous implants, standard and customized implants
*Metallic:* Titanium alloy Ti-6Al-4V	Grade V	High-load implants, customized implants
*Ceramic:* Zirconia (Y-TZP)	Fully or partially sintered	Anterior implants, experimental full implants
*Polymeric:* PEEK (polyetheretherketone)	Plain or carbon-reinforced	Experimental implants (today)

**Table 2:** Materials and technologies used for fabrication of customized dental implants.

	Materials			Manufacturing Technologies	
Category	Titanium/Ti Alloys	Zirconia (Y-TZP)	PEEK/Polymers	SM	AM
Cost	Moderate to high (higher for customized implants)	High	Moderate	Moderate (efficient for standard parts)	High (equipment and processing costs)
Indications	Standard and complex cases; high-load regions; full range of clinical scenarios	Aesthetic zones; metal-sensitive patients	Experimental/limited clinical use	Standardized implants; high precision components	Patient-specific implants; complex geometries; porous structures
Contraindications	Rare hypersensitivity; aesthetic limitations in thin gingiva	High-load posterior regions; bruxism; risk of fracture	Limited evidence; low bioactivity without modification	Complex internal geometries; customized designs	Limited availability; need for post-processing; still evolving standards
Survival Rates	Well documented: >90–95% (long-term)	Promising but slightly lower; limited long-term data	Insufficient clinical data	Comparable to conventional implants	Promising, but long-term clinical validation needed
Mechanical Properties	Excellent strength and fatigue resistance	High strength but brittle	Lower strength; elastic modulus closer to bone	High precision and surface quality	Tunable porosity and mechanical properties
Biological Performance	Excellent osseointegration	Good soft tissue response; low plaque affinity	Requires surface modification for bioactivity	Established surface treatments	Advanced surface/topography control possible
Material–technology compatibility	Fully compatible with SM and AM	Primarily compatible with SM; limited AM applications	Compatible with SM and AM (mainly experimental)	Applicable to most dense materials	Best suited for metals and polymers; limited for ceramics
Surface strategy	Established surface treatments (e.g., blasting, etching)	Limited but developing surface modifications	Requires modification to enhance bioactivity	Surface modification applied post-fabrication	Surface and porosity can be integrated during fabrication
Main Limitations	High stiffness (stress shielding); cost	Brittleness; sensitivity to defects	Lack of osteoconductivity; experimental status	Material waste; limited design flexibility	Cost; complexity; post-processing requirements

## Data Availability

No new data were created or analyzed in this study. Data sharing is not applicable to this article.

## References

[1] Elazzouni D., Alsunbul H., Almnea R., Awadh W., Alshehri A., Alhaidry H., Javed R., Khan A. (2026). Artificial Intelligence in the Synthesis and Application of Advanced Dental Biomaterials: A Narrative Review of Probabilities and Challenges. Rev. Adv. Mater. Sci..

[2] Huang Y.-C., Huang Y.-C., Ding S.-J. (2023). Primary Stability of Implant Placement and Loading Related to Dental Implant Materials and Designs: A Literature Review. J. Dent. Sci..

[3] Alazmi S. (2025). Role of Implant Diameter and Length on Success Rates: A Narrative Review. J. Pharm. Bioallied Sci..

[4] Zhang T.-M., Li X., Luo R., Gao L.-L., Wang Y.-N., Wang T., Sun M.-C., Yan Y.-B., Shen J., Li R.-X. (2025). Effects of 3D-Printed Porous Ti-6Al-4V Scaffold Pore Structure and Micro-Nano Surface Topography on the Repair of Maxillofacial Bone Defects. Mater. Des..

[5] He S., Zhu J., Jing Y., Long S., Tang L., Cheng L., Shi Z. (2024). Effect of 3D-Printed Porous Titanium Alloy Pore Structure on Bone Regeneration: A Review. Coatings.

[6] Alqutaibi A.Y., Alghauli M.A., Aljohani M.H.A., Zafar M.S. (2024). Advanced Additive Manufacturing in Implant Dentistry: 3D Printing Technologies, Printable Materials, Current Applications and Future Requirements. Bioprinting.

[7] Huang S., Wei H., Li D. (2023). Additive Manufacturing Technologies in the Oral Implant Clinic: A Review of Current Applications and Progress. Front. Bioeng. Biotechnol..

[8] Liu H., Xuan Gan M., Zhai W., Song X. (2023). Design and Additive Manufacturing of Root Analogue Dental Implants: A Comprehensive Review. Mater. Des..

[9] Li R.W.K., Chow T.W., Matinlinna J.P. (2014). Ceramic Dental Biomaterials and CAD/CAM Technology: State of the Art. J. Prosthodont. Res..

[10] Freitas B., Richhariya V., Silva M., Vaz A., Lopes S.F., Carvalho Ó. (2025). A Review of Hybrid Manufacturing: Integrating Subtractive and Additive Manufacturing. Materials.

[11] Ben Said L., Ayadi B., Alharbi S., Dammak F. (2025). Recent Advances in Additive Manufacturing: A Review of Current Developments and Future Directions. Machines.

[12] Haleem A., Javaid M., Rab S., Singh R.P., Suman R., Kumar L. (2023). Significant Potential and Materials Used in Additive Manufacturing Technologies towards Sustainability. Sustain. Oper. Comput..

[13] Ebrahimi M., Shaikh H., Rezvani Sichani H., Ramachandran R.A., Paramasivan M., Fazle Alam M., Mezzomo L., Dubey N., Mathew M.T. (2026). Additive Manufacturing for Dentistry: A Comprehensive Review of Techniques and Applications. Prog. Mater. Sci..

[14] Zakir M., Ashraf U., Tian T., Han A., Qiao W., Jin X., Zhang M., Tsoi J.K., Matinlinna J.P. (2016). The Role of Silane Coupling Agents and Universal Primers in Durable Adhesion to Dental Restorative Materials—A Review. Curr. Oral Health Rep..

[15] Armstrong M., Mehrabi H., Naveed N. (2022). An Overview of Modern Metal Additive Manufacturing Technology. J. Manuf. Process..

[16] Turek P., Zaborniak M., Grzywacz-Danielewicz K., Bałuszyński M., Lewandowski B., Kluczyński J., Daniel N. (2025). A Review of the Most Commonly Used Additive Manufacturing Techniques for Improving Mandibular Resection and Reconstruction Procedures. Appl. Sci..

[17] Pradíes G., Francisco B.M. (2023). Current Applications of 3D Printing in Dental Implantology: A Scoping Review Mapping the Evidence. Clin. Oral Implant. Res..

[18] Jiao M., Long H., Xiao B., Liang X., Lin F. (2024). Electron Beam Powder Bed Fusion Additive Manufacturing: A Comprehensive Review and Its Development in China. Addit. Manuf. Front..

[19] Teixeira Ó., Silva F.J.G., Ferreira L.P., Atzeni E. (2020). A Review of Heat Treatments on Improving the Quality and Residual Stresses of the Ti–6Al–4V Parts Produced by Additive Manufacturing. Metals.

[20] Negi S., Nambolan A.A., Kapil S., Joshi P.S., Manivannan R., Karunakaran K.P., Bhargava P. (2020). Review on Electron Beam Based Additive Manufacturing. Rapid Prototyp. J..

[21] Ali S., Deiab I., Pervaiz S. (2024). State-of-the-Art Review on Fused Deposition Modeling (FDM) for 3D Printing of Polymer Blends and Composites: Innovations, Challenges, and Applications. Int. J. Adv. Manuf. Technol..

[22] Pillai S., Upadhyay A., Khayambashi P., Farooq I., Sabri H. (2021). Dental 3D-Printing: Transferring Art from the Laboratories to the Clinics. Polymers.

[23] Acierno D., Patti A. (2023). Fused Deposition Modelling (FDM) of Thermoplastic-Based Filaments: Process and Rheological Properties—An Overview. Materials.

[24] Lüchtenborg J., Burkhardt F., Nold J., Rothlauf S., Wesemann C., Pieralli S., Kleinvogel G., Witkowski S., Spies B. (2021). Implementation of Fused Filament Fabrication in Dentistry. Appl. Sci..

[25] Kowalski J., Rylska D., Januszewicz B., Konieczny B., Cichomski M., Matinlinna J.P., Radwanski M., Sokolowski J., Lukomska-Szymanska M. (2023). Corrosion Resistance of Titanium Dental Implant Abutments: Comparative Analysis and Surface Characterization. Materials.

[26] Hossain N., Aminul M., Shakib M. (2024). Results in Chemistry Advances and Significances of Titanium in Dental Implant Applications. Results Chem..

[27] Calazans Neto J.V., Celles C.A.S., de Andrade C.S.A.F., Afonso C.R.M., Nagay B.E., Barão V.A.R. (2024). Recent Advances and Prospects in β-Type Titanium Alloys for Dental Implants Applications. ACS Biomater. Sci. Eng..

[28] Poli P.P., de Miranda F.V., Polo T.O.B., Santiago Júnior J.F., Lima Neto T.J., Rios B.R., Assunção W.G., Ervolino E., Maiorana C., Faverani L.P. (2021). Titanium Allergy Caused by Dental Implants: A Systematic Literature Review and Case Report. Materials.

[29] Zhao Q., Ueno T., Wakabayashi N. (2023). A Review in Titanium-Zirconium Binary Alloy for Use in Dental Implants: Is There an Ideal Ti-Zr Composing Ratio?. Jpn. Dent. Sci. Rev..

[30] Liu X., Chen S., Tsoi J.K.H., Matinlinna J.P. (2017). Binary Titanium Alloys as Dental Implant Materials—A Review. Regen. Biomater..

[31] Apratim A., Eachempati P., Salian K.K.K., Singh V., Chhabra S., Shah S. (2015). Zirconia in Dental Implantology: A Review. J. Int. Soc. Prev. Community Dent..

[32] Aldhuwayhi S. (2025). Zirconia in Dental Implantology: A Review of the Literature with Recent Updates. Bioengineering.

[33] Qu Y., Liu L. (2021). Zirconia Materials for Dental Implants: A Literature Review. Front. Dent. Med..

[34] Zakaria Z., Hasmady S., Shaari N., Yahaya A.Z., Yap B.K. (2019). A Review on Recent Status and Challenges of Yttria Stabilized Zirconia Modification to Lowering the Temperature of Solid Oxide Fuel Cells Operation. Int. J. Energy Res..

[35] Shahmiri R., Standard O.C., Hart J.N., Sorrell C.C. (2018). Optical Properties of Zirconia Ceramics for Esthetic Dental Restorations: A Systematic Review. J. Prosthet. Dent..

[36] Koller M., Steyer E., Theisen K., Stagnell S., Jakse N., Payer M. (2020). Two-Piece Zirconia versus Titanium Implants after 80 Months: Clinical Outcomes from a Prospective Randomized Pilot Trial. Clin. Oral Implant. Res..

[37] Roehling S., Schlegel K.A., Woelfler H., Gahlert M. (2018). Performance and Outcome of Zirconia Dental Implants in Clinical Studies: A Meta-Analysis. Clin. Oral Implant. Res..

[38] Cattani-Lorente M.A., Scherrer S.S., Ammann P., Jobin M., Wiskott A.H.W. (2011). Low Temperature Degradation of a Y-TZP Dental Ceramic. Acta Biomater..

[39] Hashim D., Cionca N., Courvoisier D.S., Mombelli A. (2016). A Systematic Review of the Clinical Survival of Zirconia Implants. Clin. Oral Investig..

[40] Panayotov I.V., Orti V., Cuisinier F., Yachouh J. (2016). Polyetheretherketone (PEEK) for Medical Applications. J. Mater. Sci. Mater. Med..

[41] Kurtz S.M., Devine J.N. (2007). PEEK Biomaterials in Trauma, Orthopedic, and Spinal Implants. Biomaterials.

[42] Park S., Jung T.-G. (2024). Surface Modification of Polyetheretherketone (PEEK) Intervertebral Fusion Implant Using Polydopamine Coating for Improved Bioactivity. Bioengineering.

[43] Buck E., Li H., Cerruti M. (2020). Surface Modification Strategies to Improve the Osseointegration of Poly(Etheretherketone) and Its Composites. Macromol. Biosci..

[44] Zakir M., Laiho T., Granroth S., Kukk E., Chu C.H., Tsoi J.K.-H., Matinlinna J.P. (2022). A Novel Dual Surface Modification on Titanium in Dental Use: Characterization and Topography. Surf. Interface Anal..

[45] Zakir M., Laiho T., Granroth S., Kukk E., Tsoi J.K.H., Chu C.H., Matinlinna J.P. (2023). A New Adhesion Concept Based on a Dual Surface Modification for Resin Ti Adhesion. Int. J. Adhes. Adhes..

[46] Wu Y., Wan K., Lu J., Yuan C., Cui Y., Duan R., Yu J. (2025). Research Progress on Surface Modification of Titanium Implants. Coatings.

[47] Kim Y.-W. (2010). Surface Modification of Ti Dental Implants by Grit-Blasting and Micro-Arc Oxidation. Mater. Manuf. Process..

[48] Sayin Ozel G., Inan O., Secilmis Acar A., Alniacik Iyidogan G., Dolanmaz D., Yildirim G. (2021). Stability of Dental Implants with Sandblasted and Acid-Etched (SLA) and Modified (SLActive) Surfaces during the Osseointegration Period. J. Dent. Res. Dent. Clin. Dent. Prospect..

[49] Schünemann F.H., Galárraga-Vinueza M.E., Magini R., Fredel M., Silva F., Souza J.C.M., Zhang Y., Henriques B. (2019). Zirconia Surface Modifications for Implant Dentistry. Mater. Sci. Eng. C Mater. Biol. Appl..

[50] Schupbach P., Glauser R., Bauer S. (2019). Al_2_O_3_ Particles on Titanium Dental Implant Systems Following Sandblasting and Acid-Etching Process. Int. J. Biomater..

[51] Heikkinen T.T., Matinlinna J.P. (2009). Dental Zirconia Adhesion with Silicon Compounds Using Some Experimental and Conventional Surface Conditioning Methods. Silicon.

[52] Heikkinen T.T., Lassila L.V.J., Matinlinna J.P., Vallittu P.K. (2007). Effect of Operating Air Pressure on Tribochemical Silica-Coating. Acta Odontol. Scand..

[53] Yan Guo C., Tin Hong Tang A., Pekka Matinlinna J. (2012). Insights into Surface Treatment Methods of Titanium Dental Implants. J. Adhes. Sci. Technol..

[54] Ho B.J., Tsoi J.K.-H., Liu D., Lung C.Y.-K., Wong H.-M., Matinlinna J.P. (2015). Effects of Sandblasting Distance and Angles on Resin Cement Bonding to Zirconia and Titanium. Int. J. Adhes. Adhes..

[55] Jiang M., Sun Y., Nie Z., Palin W.M., Qian L., Matinlinna J.P., Hu L., Zhang Z. (2025). Cavitation Erosion: An Efficient Method for Promoting Osteogenesis and Immunomodulation of Titanium Surface. Dent. Mater..

[56] Tuikampee S., Chaijareenont P., Rungsiyakull P., Yavirach A. (2024). Titanium Surface Modification Techniques to Enhance Osteoblasts and Bone Formation for Dental Implants: A Narrative Review on Current Advances. Metals.

[57] Iwaya Y., Machigashira M., Kanbara K., Miyamoto M., Noguchi K., Izumi Y., Ban S. (2008). Surface Properties and Biocompatibility of Acid-Etched Titanium. Dent. Mater. J..

[58] Panda S., Kazama M., Kawai T., Biswas C.K., Paul S. (2022). Controlled Surface Modification of Ti6Al4V Using Biomimetic Mineralization via Thermo-Chemical Route Improves Bioactivity. Ceram. Int..

[59] Mondal S., Park S., Choi J., Vu T.T.H., Doan V.H.M., Vo T.T., Lee B., Oh J. (2023). Hydroxyapatite: A Journey from Biomaterials to Advanced Functional Materials. Adv. Colloid Interface Sci..

[60] Su Y., Cockerill I., Zheng Y., Tang L., Qin Y.-X., Zhu D. (2019). Biofunctionalization of Metallic Implants by Calcium Phosphate Coatings. Bioact. Mater..

[61] Zakir M., Chu C.H., Tsoi J.K.H., Daood U., Matinlinna J.P. (2023). A Novel Combined Silica-Coating and Etching Protocol for Titanium for Improved Adhesion. Silicon.

[62] Durdu S., Usta M., Berkem A. (2016). Bioactive Coatings on Ti6Al4V Alloy Formed by Plasma Electrolytic Oxidation. Surf. Coat. Technol..

[63] Lung C.Y.K., Abdalla M.M., Chu C.H., Yin I., Got S.-R., Matinlinna J.P. (2021). A Multi-Element-Doped Porous Bioactive Glass Coating for Implant Applications. Materials.

[64] Kei Lung C.Y., Khan A.S., Zeeshan R., Akhtar S., Chaudhry A.A., Matinlinna J.P. (2023). An Antibacterial Porous Calcium Phosphate Bilayer Functional Coatings on Titanium Dental Implants. Ceram. Int..

